# Hamstring tendon autografts and allografts show comparable clinical outcomes and knee stability after anterior cruciate ligament reconstruction in patients over fifty years old with no signs of osteoarthritis progression

**DOI:** 10.1007/s00264-022-05465-3

**Published:** 2022-06-08

**Authors:** Riccardo D’Ambrosi, Riccardo Giorgino, Katia Corona, Tarun Jaykumar, Ilaria Mariani, Nicola Ursino, Laura Mangiavini, Raju Vaishya

**Affiliations:** 1 IRCCS Orthopedic Institute Galeazzi, Milan, Italy; 2 Department of Biomedical Sciences for Health, University of Milan, 20133 Milan, Italy; 3 Residency Program in Orthopedics and Traumatology, University of Milan, 20122 Milan, Italy; 4 Department of Medicine, Health Sciences “Vincenzo Tiberio,” University of Molise, Campobasso, Italy; 5 Sunshine Hospital, Hyderabad, India; 6 Institute, for Maternal and Child Health IRCCS Burlo Garofolo, Trieste, Italy; 7 Indraprastha Apollo Hospitals, New Delhi, India

**Keywords:** ACL reconstruction, Hamstring, Allograft, Autograft, Over 50, Osteoarthritis progression

## Abstract

**Purpose:**

The aim of this study is to compare the functional outcomes and osteoarthritis (OA) progression after anterior cruciate ligament (ACL) reconstruction with either hamstring autografts or allografts in people over 50.

**Methods:**

The clinical records of two consecutive cohorts of 61 cases in total over 50 years of age, undergoing ACL reconstruction, were included. The first cohort consisted of 29 allografts; the second cohort consisted of 32 autologous hamstring tendon grafts. The cases were evaluated pre- (T_0_) and post-operatively at six months (T_1_), 12 months (T_2_) and 24 months (T_3_). Clinical examination included the Lachman test, pivot shift test and objective (Objective IKDC [The International Knee Documentation Committee] score) and subjective clinical scores (Subjective IKDC score, Lysholm score and Tegner activity score). The degree of OA was evaluated using the Kellgren-Lawrence system at the time of the final follow-up, compared to the pre-operative condition.

**Results:**

No pre-operative difference was found between the two groups (*p* > 0.05). No statistical difference was noted between the two groups at each follow-up (*p* > 0.05). At the final follow-up, both the groups significantly improved statistically in all the clinical and functional scores (*p* < 0.05). In both groups, one graft re-rupture was noted. No progression of OA was noted in both groups at final follow-up (*p* > 0.05).

**Conclusion:**

The graft choice does not influence the outcomes two years after ACL reconstruction in people over 50; thus, both treatments help in regaining knee stability with no signs of OA progression.

**Registration:**

Researchregistry7539–www.researchregistry.com.

## Introduction

Anterior cruciate ligament (ACL) injury is one of the most common ligament injuries of the knee in pivot-sports players. Until two decades ago, ACL reconstruction was discouraged in people over 50 owing to post-operative knee stiffness, residual instability and progression of osteoarthritis (OA). While non-operative management is noted to have acceptable functional outcomes in older patients, they need to sacrifice high-level sports and recreations [1, 2]. With an increase in the life expectancy and functional demands of the patients and a predicted doubling of the middle-aged population by the year 2050, despite the surgical risks, patients prefer surgery for a faster return to sports and other activities. Recent literature also supports favourable outcomes in older patients with proper indications [3, 4].

The choice of an ACL graft, however, remains controversial. Allografts have the advantage of lesser post-operative pain, faster recovery and better cosmetic effects but are much more expensive than the autografts, are not widely accessible and carry more risk of disease transmission. Hamstring grafts, on the contrary, have better outcomes and acceptability in younger age groups as well as lesser graft failure rates [4, 5] and do not invite any extra expenditure or pose the risk of disease transmission.

The purpose of this study is to evaluate and compare the functional outcomes and progression of OA after ACL reconstruction using either autologous hamstring or allografts in people over 50.

## Materials and methods

The Institutional Review Board’s ethical approval and the participants’ informed consent were obtained before starting the study, which was registered in the Research Registry (researchregistry7539). The study was conducted according to the Strobe Checklist [6].

The prospective clinical records of two consecutive cohorts with a total of 61 patients over 50 years undergoing ACL reconstruction were included in a single-centre study.

The first cohort (ALL group) consisted of 29 patients over 50 who underwent ACL reconstruction using an allograft (tibialis anterioris or posterioris); the second cohort had 32 patients over 50 who underwent ACL reconstruction with autologous hamstring tendon (HT group).

The inclusion criteria were a unilateral primary ACL tear, sports participation before the injury, Kellgren-Lawrence grade 0 to 2, patellofemoral OA grade 0–2 evaluated during arthroscopy with Outerbridge classification and a pre-operative positive Lachman test [7, 8]. Cases with recurrent ACL tears, a history of previous surgery on the affected knee (meniscectomy or meniscal suture excepted), multi-ligament knee injuries, Kellgren-Lawrence grade 3 and 4, patellofemoral OA grades 3–4 evaluated during arthroscopy with Outerbridge classification rheumatoid arthritis and those that necessitated an extra-articular procedure or posterior cruciate ligament (PCL) reconstruction were excluded.

Concomitant meniscal surgery (meniscectomy) was not considered an exclusion criterion. All the procedures were performed by two of the authors experienced in the use of hamstring and allograft for ACL reconstruction, respectively, and all the patients followed a similar post-operative rehabilitation protocol [9, 10].

### Clinical evaluation

The patients were evaluated pre- (T_0_) and six months (T_1_), 12 months (T_2_) and 24 months (T_3_) post-operatively by a clinician not involved in the surgery. Clinical examination included the Lachman test, pivot shift test, and objective (Objective IKDC [The International Knee Documentation Committee] score) and subjective clinical scores (Subjective IKDC score, Lysholm score and Tegner activity score). The evaluated parameters of the study groups were compared at all follow-up intervals. The post-operative complications of all the patients were recorded [11–13].

### Radiographic assessment

The degree of OA was determined based on the Kellgren-Lawrence grading system on weight-bearing anteroposterior and lateral radiographs at the time of the final follow-up, compared to the pre-operative condition. Radiographic assessment for OA was performed by an orthopaedic sports physician who was not part of the operative team [7].

### Surgical technique

The choice of which graft to use was based on the surgeon’s experience. In both groups, an ACL reconstruction was performed through an anteromedial portal in supine position, with a padded pneumatic tourniquet applied to the thigh. Two posts were then attached to the surgical table, the first lateral to the proximal thigh and the second as a foot roll meant to maintain 90° of knee flexion. If the meniscal injury was stable, it was left that way; if it was unstable, a hyperselective meniscectomy of the injured area was performed. Hamstring graft harvesting was then performed through a 2-cm skin incision on the upper medial tibia [14].

### Post-operative rehabilitation

Isometric quadriceps contractions were started on the second day after surgery. Range of motion (ROM) up to 0–90° was permitted during the first post-operative week and 0–120° during the second, followed by a progressive increase until full ROM at six weeks. Progressive weight-bearing was allowed as tolerated, starting the second day after surgery. An activity return was allowed at three months, six months and eight to nine months post-operatively to non-pivoting and non-contact sports, pivoting and non-contact sports, and contact and pivoting sports, respectively [15].

### Sample size

An estimated sample of 58 subjects, 29 for each group, was required to compare the subjective IKDC between allograft and autograft with a two-sided *t* test, assuming a mean difference of 15 and a standard deviation (SD) of 20 for both the groups as well as a 5% alpha and an 80% power. Given the same parameters, this sample also had a 99% power to detect a difference among follow-up measurements. Additional subjects were recruited to ensure statistical significance in case of adverse events [16].

### Statistical analysis

A summary of the statistics is presented as mean and SD or absolute frequency and percentage. After testing the distribution of continuous variables, a *t* test or a chi-square or a Fisher exact test for categorical variables was performed to assess the pre-operative differences between the ALL and HT groups.

To test the possible score change by time in each group (T_0_: pre-operative; T_1_: six months follow-up measure; T_2_: 12 months follow-up measure; T_3_: 24 months follow-up measure), a mixed model was employed since it allows the consideration of the correlations among repeated measures and the testing of the covariance structure. The compound symmetry, autoregressive, Toepliz, Huynh–Feldt and unstructured covariance structures were tested, and the unstructured covariance structure was adjudged the best, using the likelihood ratio test and the Akaike information criterion. In case of ordinal scores, a Wilcoxon signed-rank test was performed. The Bonferroni adjustment was applied for multiple comparisons. To assess the difference in new injuries between the groups, a Fisher exact test was performed, and the correlations among variables were analysed, according to variable distribution, with the Pearson or Spearman correlations. All tests were two-sided, and *p* values less than 0.05 were considered statistically significant. Statistical analyses were conducted in R version 4.1.1 and SAS/STAT 9.3.

## Results

A total of 61 patients were included in the study, with 29 in the ALL group and 32 in the HT group. No pre-operative differences were found between the two groups (*p* > 0.05). No differences were found between the groups regarding associated meniscal injuries (Table 1). The only significant difference was in the tourniquet time, which was significantly lower in the ALL group (41.72 versus 68.88 min; *p* < 0.001). Detailed results are presented in Table 2.

**Table 1:** Meniscal Injuries incidence in both groups

	Allograft *N* = 29 ***n* (%)**	Hamstrings *N* = 32 ***n* (%)**	*p* value
MM	8 (27.6)	9 (28.1)	0.963
ML	4 (13.8)	5 (15.6)	0.980
Total	12 (41.4)	14 (43.8)	0.852

*MM* medial meniscus injury, *ML* lateral meniscus injury

**Table 2:** The descriptive characteristics of the study groups

	Allograft *N* = 29 *n* (%)	Hamstrings *N* = 32 *n* (%)	*p* value
**Sociodemographic characteristics**			
Sex			
Female	10 (34.5)	16 (50.0)	0.335
Male	19 (65.5)	16 (50.0)	
Age	53.83 (3.19)	54.09 (3.70)	0.766
**Clinical characteristics**			
Tourniquet time	41.72 (8.47)	68.88 (8.47)	< 0.001*
Knee			
Right	10 (34.5)	18 (56.2)	0.148
Left	19 (65.5)	14 (43.8)	
**Pre surgery scores**			
Lachmann test			
1	3 (10.3)	2 (6.2)	0.810
2	14 (48.3)	15 (46.9)	
3	12 (41.4)	15 (46.9)	
Pivot Shift test			
1	3 (10.3)	7 (21.9)	0.440
2	18 (62.1)	16 (50.0)	
3	8 (27.6)	9 (28.1)	
Objective IKDC			
B	1 (3.4)	1 (3.1)	0.993
C	13 (44.8)	14 (43.8)	
D	15 (51.7)	17 (53.1)	
Subjective IKDC	49.52 (16.35)	49.62 (15.94)	0.979
Lysholm score	70.38 (15.34)	70.38 (15.42)	0.999
Tegner score	4.83 (1.54)	4.91 (1.71)	0.851

^*^ Statistical significant value (*p* < 0.05)

IKDC = The International Knee Documentation Committee

### Categorical indices (Lachman test, pivot shift test, objective IKDC)

Both groups showed significant improvements at final follow-up in the Lachman test, pivot shift test and objective IKDC score when compared to the pre-operative value (*p* < 0.05). No significant difference was found between the two groups (*p* > 0.05). The detailed results are reported in Table 3.

**Table 3:** Clinical comparison between the two groups for *Categorical Indexes* (*Lachmann test*, *Pivot Shift Test*, *Objective IKDC*)

Allograft *N* = 29		Hamstrings *N* = 32	**Group comparison**	**Time comparison** ***Adjusted p-value***						
***n (%)***		***n (%)***	***p* value**	Allograft				Hamstrings		
**Lachmann test**										
**T**_**0**_										
1	3 (10.3)	2 (6.2)	0.804		T_0_	T_1_	T_2_	T_0_	T_1_	T_2_
2	14 (48.3)	15 (46.9)		T_1_	< 0.001*	-		< 0.001*	–	
3	12 (41.4)	15 (46.9)		T_2_	< 0.001*	NA	–	< 0.001*	NA	–
**T**_**1**_				T_3_	< 0.001*	NA	NA	< 0.001*	NA	NA
0	29 (100.0)	32 (100.0)	NA							
**T**_**2**_										
0	29 (100.0)	32 (100.0)	NA							
**T**_**3**_										
0	29 (100.0)	32 (100.0)	NA							
**Pivot shift test**										
**T**_**0**_										
1	3 (10.3)	7 (21.9)	0.440		T_0_	T_1_	T_2_	T_0_	T_1_	T_2_
2	18 (62.1)	16 (50.0)		T_1_	< 0.001*	-		< 0.001*	-	
3	8 (27.6)	9 (28.1)		T_2_	< 0.001*	> 0.999	–	< 0.001*	> 0.999	–
**T**_**1**_				T_3_	< 0.001*	> 0.999	> 0.999	< 0.001*	> 0.999	> 0.999
0	26 (89.7)	25 (78.1)	0.307							
1	3 (10.3)	7 (21.9)								
**T**_**2**_										
0	26 (89.7)	24 (77.4)	0.355							
1	3 (10.3)	7 (22.6)								
**T**_**3**_										
0	26 (92.9)	26 (83.9)	0.428							
1	2 (7.1)	5 (16.1)								
**Objective IKDC**										
**T**_**0**_										
B	1 (3.4)	1 (3.1)	> 0.999		T_0_	T_1_	T_2_	T_0_	T_1_	T_2_
C	13 (44.8)	14 (43.8)		T_1_	< 0.001*	-		< 0.001*	–	
D	15 (51.7)	17 (53.1)		T_2_	< 0.001*	> 0.999	–	< 0.001*	> 0.999	–
**T**_**1**_				T_3_	< 0.001*	0.432	> 0.999	< 0.001*	> 0.999	> 0.999
A	22 (75.9)	24 (75.0)	> 0.999							
B	7 (24.1)	8 (25.0)								
**T**_**2**_										
A	24 (82.8)	23 (74.2)	0.623							
B	5 (17.2)	8 (25.8)								
**T**_**3**_										
A	25 (89.3)	25 (80.6)	0.477							
B	3 (10.7)	6 (19.4)								

^*^ Statistical significant value (*p* < 0.05)

IKDC = The International Knee Documentation Committee

**Fig. 1:**
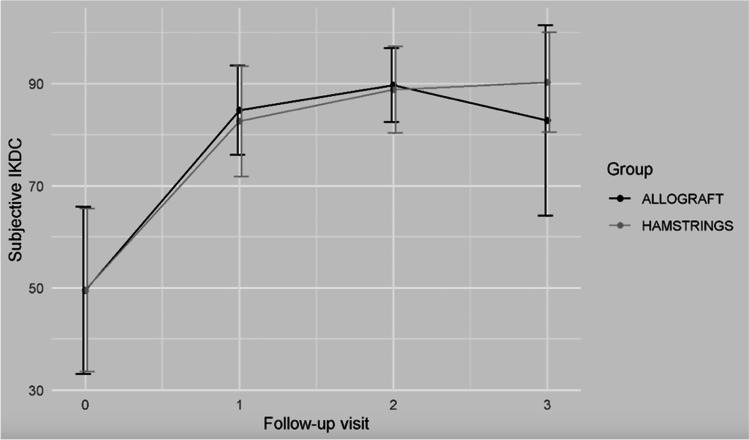
The trend in the subjective IKDC for the two groups

Figure 1 reports the trend in objective IKDC.

#### Continuous indices (Tegner, subjective IKDC, Lysholm score and Range of Motion)

No statistical differences were found between the two groups at each follow-up (*p* > 0.05).

**Fig. 2:**
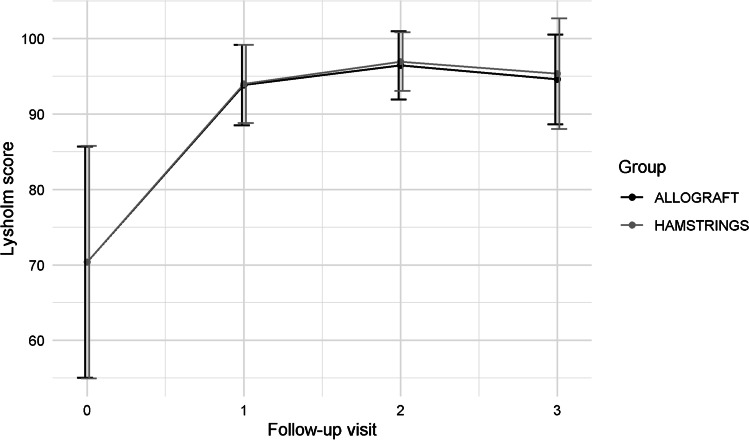
The trend in the Lysholm score for the two groups

**Fig. 3:**
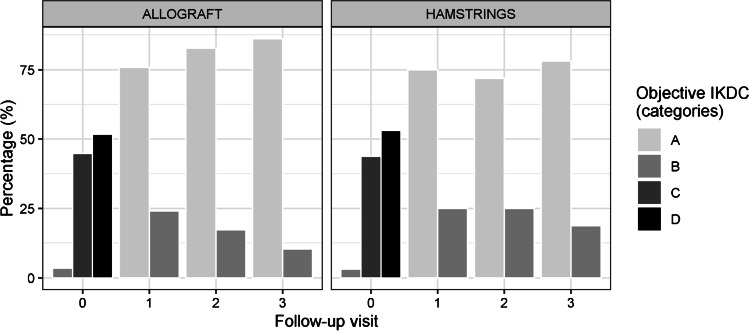
The distribution of objective IKDC scores for the two groups

**Fig. 4:**
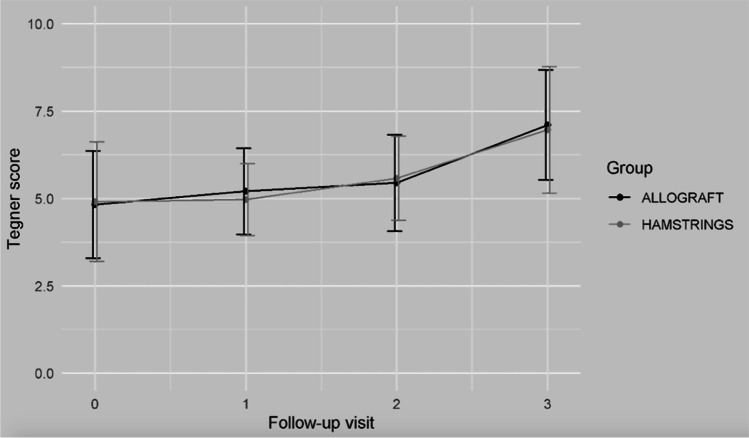
The trend in the Tegner score for the two groups

At the final follow-up, both the groups showed a statistically significant improvement in all the continuous indices (*p* < 0.05). The detailed results are reported in Table 4. Figures 2, 3 and 4 show the trends in Tegner, subjective IKDC and Lysholm scores.

**Table 4:** Clinical comparison between the two groups for *Continuous Indexes* (*Lachmann test*, *Pivot Shift Test*, *Objective IKDC*)

Allograft *N* = 29		Hamstrings *N* = 32	**Group comparison**	**Time comparison** ***Adjusted p-value***					
***Mean* ± *SD***		***Mean* ± *SD***	***p-value***	Allograft			Hamstrings		
**Tegner score**									
T_0_	5.19 ± 1.55	4.75 ± 1.81	0.329	T_0_	T_1_	T_2_	T_0_	T_1_	T_2_
T_1_	3.15 ± 1.29	3.03 ± 0.93	0.676	> 0.999	–		> 0.999	–	
T_2_	3.92 ± 1.09	3.81 ± 1.03	0.694	0.589	> 0.999	–	0.337	0.064	-
T_3_	5.27 ± 1.15	5.22 ± 1.21	0.872	< 0.001*	< 0.001*	0.001*	< 0.001*	< 0.001*	0.008*
**Subjective IKDC**									
T_0_	49.00 ± 11.76	47.72 ± 17.18	0.748	T_0_	T_1_	T_2_	T_0_	T_1_	T_2_
T_1_	57.00 ± 13.64	57.28 ± 12.06	0.934	< 0.001*	-		< 0.001*	–	
T_2_	71.50 ± 10.60	71.19 ± 13.28	0.923	< 0.001*	0.185	–	< 0.001*	0.114	-
T_3_	81.77 ± 9.26	84.75 ± 10.76	0.269	< 0.001*	> 0.999	0.274	< 0.001*	0.044*	> 0.999
**Lysholm score**									
T_0_	66.81 ± 18.31	63.44 ± 23.01	0.547	T_0_	T_1_	T_2_	T_0_	T_1_	T_2_
T_1_	77.62 ± 17.94	80.56 ± 15.49	0.505	< 0.001*	–		< 0.001*	–	
T_2_	89.31 ± 9.69	90.72 ± 7.78	0.541	< 0.001*	0.297	–	< 0.001*	0.101	-
T_3_	93.73 ± 4.77	94.78 ± 5.97	0.470	< 0.001*	> 0.999	> 0.999	< 0.001*	> 0.999	0.892
**Range of motion (°)**									
T_1_	145.69 ± 2.21	146.56 ± 2.35	0.142	T_0_	T_1_	T_2_	T_0_	T_1_	T_2_
T_2_	146.03 ± 2.06	146.45 ± 2.31	0.464	NA	0.483	–	NA	> 0.999	-
T_3_	146.25 ± 2.20	146.94 ± 2.48	0.268	NA	0.246	0.962	NA	> 0.999	0.235

^*^Statistical significant value (*p* < 0.05)

IKDC = The International Knee Documentation Committee

### Osteoarthritis assessment

The mean pre-operative Kellgren-Lawrence grade was 1.51 ± 0.51 for ALL and 1.5 ± 0.5 for HT (*p* > 0.05). At final follow-up, both groups showed a non-significant worsening (*p* > 0.05), with no differences between the two groups (*p* > 0.05) (ALL: *1.79* ± 0.62; ALL: 1.75 ± 0.57). The detailed results are reported in Table 5.

**Table 5:** Pre-operative and final follow-up Kellgren-Lawrence grading system in both groups

	Allograft *N* = 29 Mean ± SD	Hamstrings *N* = 32 Mean ± SD	Group comparison *p* value	Pre-post comparison	
				Allograft *p* value	Hamstrings *p* value
Kellgren-Lawrence score					
Pre-operative	1.51 ± 0.51	1.5 ± 0.5	0.895	0.069	0.068
Final follow-up	1.79 ± 0.62	1.75 ± 0.57	0.778		

### Failures

In both the groups, one graft re-rupture was reported: at 24 months in the ALL group and 12 months in the HT group (*p* = 0.944).

### Correlations

**Fig. 5:**
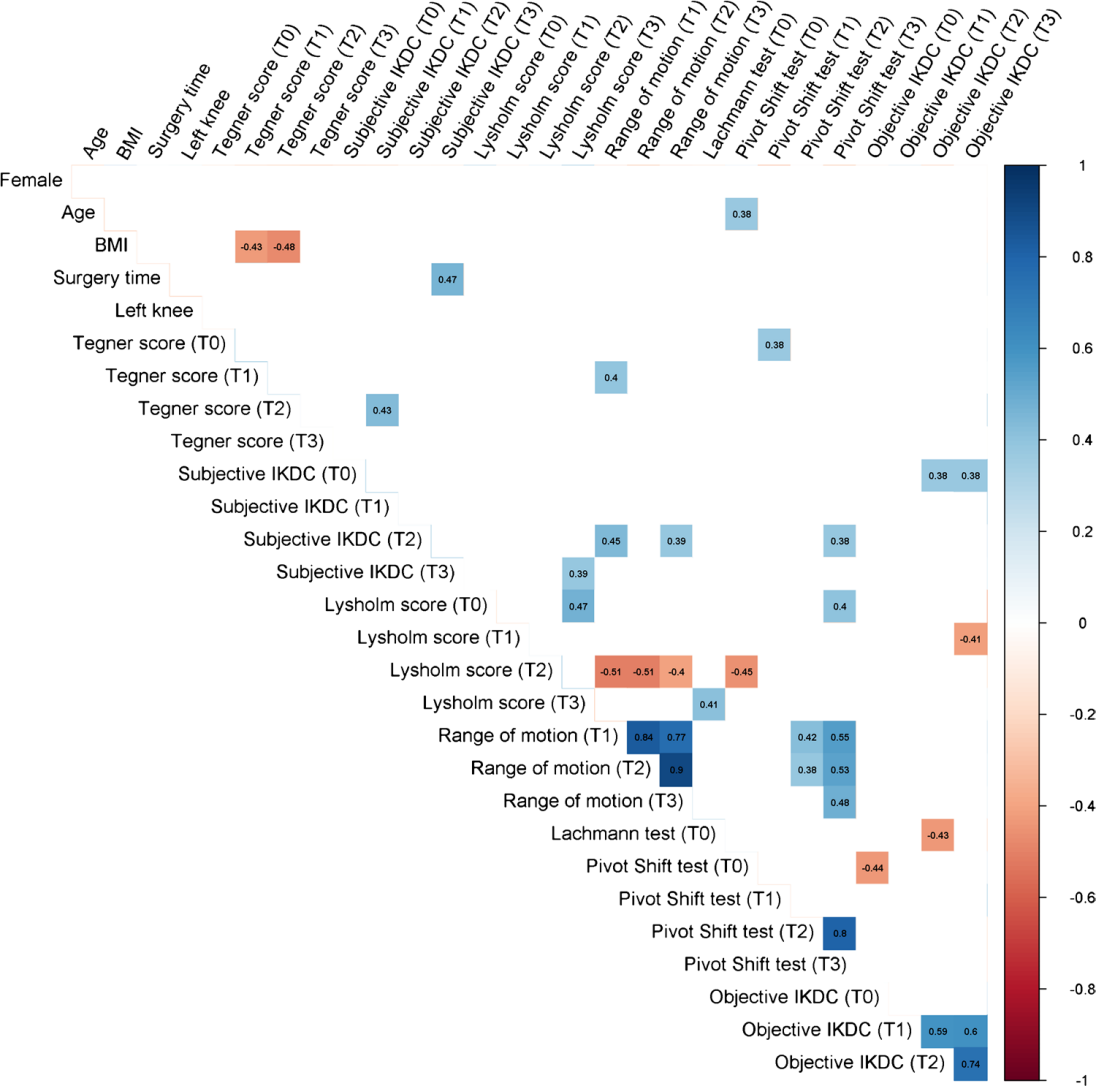
Statistically significant correlations for the ALL group

**Fig. 6:**
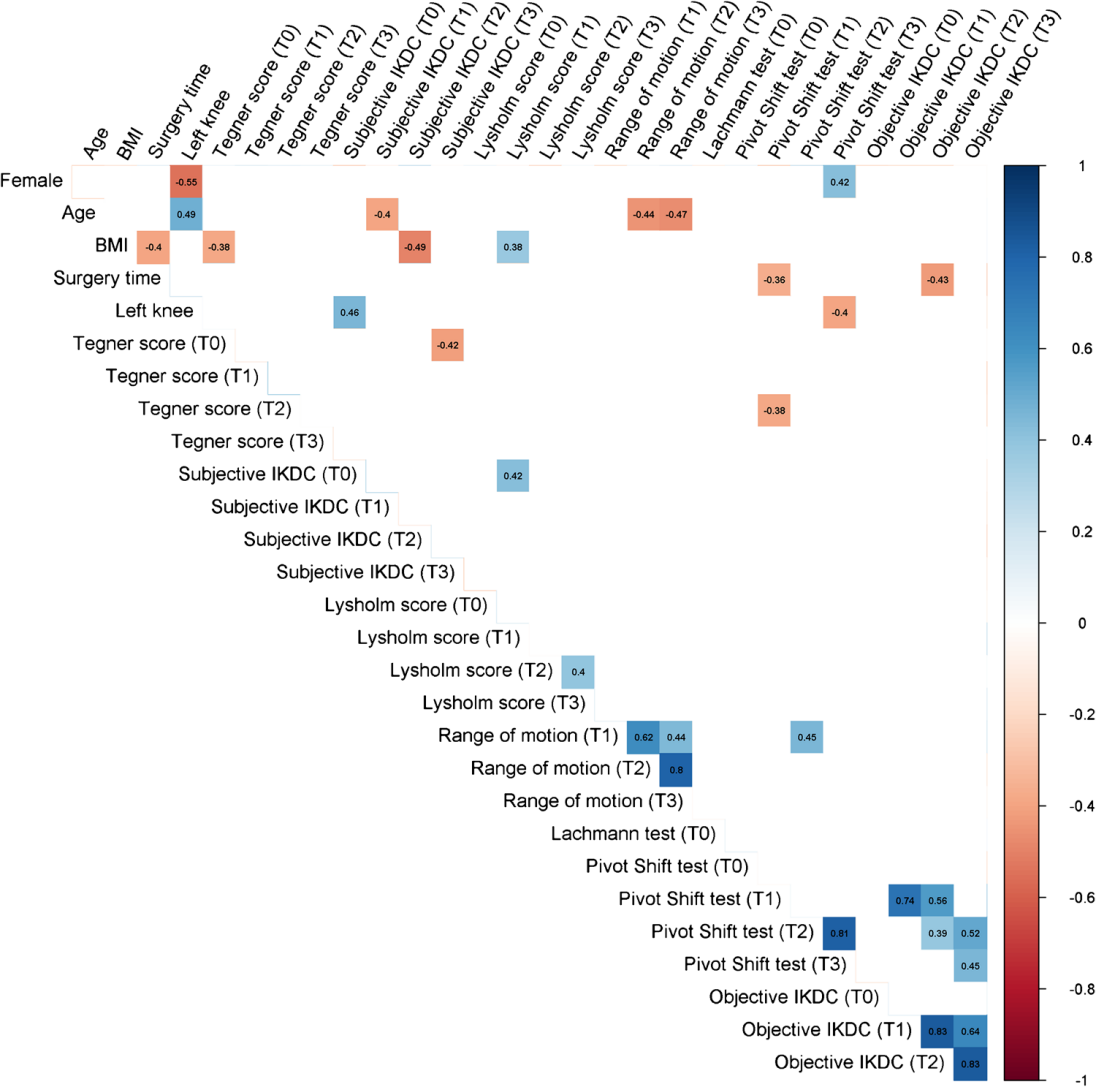
Statistically significant correlations for the HT group

The statistically significant correlations are reported in Figs. 5 and 6.

## Discussion

Our results show no pre-operative differences between the ALL group and the HT group apart from the tourniquet time, which was lower in the ALL group. Cases in both the groups showed significant improvements at the final follow-up in the Lachman test, pivot shift test and objective IKDC score, when compared to the pre-operative values. No statistical difference was found between the two groups at each follow-up. At the final follow-up, both the groups showed a statistically significant improvement in all the continuous indices without OA progression.

A systematic review published in 2019 reported OA progression after ACL reconstruction in patients over 50 years old, reporting radiographic signs of progression of OA in six studies, in which severe signs of degeneration (grade 3 or 4 according to Kellgren–Lawrence or Ahlbäck classification) increased from four out of 216 knees (1.9%) before surgery to 28 out of 187 knees (15%) following ACL reconstruction, after a mean period of follow-up ranging from 32 to 64 months [3].

Whether people over 50 with ACL injuries should be considered for a surgical reconstruction has long been debated. A recent study by Ventura et al. retrospectively evaluated outcomes after ACL reconstructions with hamstring autografts in cases over 50 years of age [17]. Their results with a mean follow-up of 4.4 years showed favourable outcomes concerning knee stability and patient satisfaction after an ACL reconstruction in most cases, with increased Lysholm, IKDC and Tegner scores and also in clinical evaluation and instrumented laxity tests. Furthermore, the degree of OA did not statistically increase in their follow-up.

In a multi-centre, prospective, non-randomised follow-up study by Panisset et al., 228 patients over 50 years and 130 patients under 40 were comparatively evaluated [2]. Besides differences in terms of associated injuries such as meniscal tears and cartilage injuries, which presented more in the older patient group, the functional results of ACL reconstruction were equivalent in both the groups, with identical rates of late complications. This treatment was, therefore, justified and found effective, and they recommended that an ACL reconstruction should also be offered to active individuals over 50 with symptomatic instability.

Associated lesions could be a useful tool in selecting the suitable candidate for surgical ACL reconstruction, as suggested by Fayard et al. [18]. This study focused on detecting possible risk factors and identified medial tibiofemoral OA and medial meniscal injury in pre-operative explosive pivot-shift. It is, therefore, essential to carry out a rigorous selection of patients, before lesions of the medial meniscus can occur.

In all cases of unstable meniscal lesion, we have decided to perform a meniscectomy and not a meniscal suture, because a recent study has shown that in patients over 40 years old the two procedures have comparable results; moreover, the risk of failure after meniscal suture in patients over 40 years old can be more than 20% [19, 20].

Nowadays, patients over 50 have a higher quality of life and, above all, a higher functional demand. Although not comparable to the requests of sports athletes, these individuals expect to return to sports and, primarily, to an active and performing working life; furthermore, they want to do it as soon as possible. Ovigue et al. evaluated the return to sports after more than two years of follow-up in patients over 50 following ACL reconstructions with autologous hamstring grafts [1]. The analysis revealed significant improvement in IKCD, Lysholm, KOOS and Tegner scores, and most cases returned to sports at the same level they had before the injury. Particularly, the Tegner score before the accident proved to be a positive factor that influenced their return to the pre-injury level of the sport. Surgical treatment now seems a safe and reliable treatment option, identifying the most suitable surgical technique for a patient over 50 is imperative.

If patients over 50 require that they return to their activities as soon as possible, an ACL reconstruction with the allograft technique would seem an attractive option, as it is performed in a shorter surgical time and is associated with a quicker post-operative recovery. Krupa et al. compared the reconstruction with autologous hamstring and with allograft in terms of costs of surgery, post-operative pain, post-operative complications, time required to return to work or other similar physical activity and aesthetic outcome [4]. The allograft technique presented a shorter surgery time, less post-operative pain, fewer local complications and a better aesthetic outcome pertaining to the scar; principally, it required a shorter time before a return to office work was possible, albeit with a higher economic cost.

It is crucial to reflect on the kind of quality that can be expected from the removal of autologous tendons in a 50-year-old subject and the possible degenerative phenomena related to the patient’s age. The literature comparing autografts and allografts showed similar results in age-matched adult populations. Brown et al.’s study highlighted the pros and cons of autografts and allografts [21]. While several authors have discussed the effects of recommending allograft reconstruction in young subjects, the best surgical technique for elderly patients still remains contentious [22, 23]. In general, it has been proved that allografts have comparable outcomes if the grafts are processed correctly and without irradiation or aggressive chemical treatment [21]. Furthermore, a systematic review has revealed that allografts from younger donors should be preferred, and grafts subjected to high doses of radiation and chemical processes or numerous freeze–thaw cycles should be avoided [24].

To evaluate the clinical outcomes and knee stability in ACL reconstruction in patients over 50, it is essential to reflect on the relationship between surgery and OA [3]. It is always difficult to accurately determine whether the level of OA is part of the natural history of cartilage degeneration or a consequence of surgery. Possible joint injuries during surgery, pre-surgery trauma, inflammatory stress, prolonged decision time before undergoing surgery, patient’s age and alteration of joint mechanics have been hypothesised as the potential aetiopathological mechanisms [25–27]. A protective factor against the development of OA with a broad consensus is the preservation of the medial meniscus [28, 29]. Analysing these aspects, some authors conclude that ACL reconstruction in patients over 50 offers good results and that age itself does not contraindicate ACL surgery [3].

These findings inform shared decision-making and can help surgeons manage ACL injuries in a relatively older population regardless of the type of graft choice.

The present study has some limitations. Since this study was done in a high-volume tertiary referral hospital, its findings may not be generalisable to low-volume institutions where ACL surgeries are infrequent. Moreover, this study followed patients for only one to two years post-surgically; however, patient activity and knee stability may change beyond this period. Additionally, the two groups were large, and a power analysis was performed to ensure sample size, which again reduces the generalisability of the study. In the future, more studies involving long-term follow-up of patients who have undergone supervised physiotherapy treatments should be considered.

## Conclusions

The ACL graft choice does not influence the clinical and functional outcomes two years after reconstruction in cases over 50 years of age. The ACL reconstruction allows these individuals to regain knee stability with no signs of OA progression. The allografts and hamstring autografts demonstrated similar functional and objective results, although the surgical time for the allograft is shorter.

## Data Availability

Raw data have been submitted as supplementary material to the Journal.

## References

[1] Ovigue J, Bouguennec N, Graveleau N (2020). Arthroscopic anterior cruciate ligament reconstruction is a reliable option to treat knee instability in patients over 50 years old. Knee Surg Sports Traumatol Arthrosc.

[2] Panisset JC, Gonzalez JF, de Lavigne C, Ode Q, Dejour D, Ehlinger M, Fayard JM, Lustig S, French Arthroscopic Society (2019). ACL reconstruction in over-50 year-olds: comparative study between prospective series of over-50 year-old and under-40 year-old patients. Orthop Traumatol Surg Res.

[3] Costa GG, Grassi A, Perelli S, Agrò G, Bozzi F, Lo Presti M, Zaffagnini S (2019). Age over 50 years is not a contraindication for anterior cruciate ligament reconstruction. Knee Surg Sports Traumatol Arthrosc.

[4] Krupa S, Reichert P (2020). Factors influencing the choice of graft type in ACL reconstruction: allograft vs autograft. Adv Clin Exp Med.

[5] Bowman EN, Limpisvasti O, Cole BJ, ElAttrache NS (2021). Anterior cruciate ligament reconstruction graft preference most dependent on patient age: a survey of United States surgeons. Arthroscopy.

[6] Cuschieri S (2019). The STROBE guidelines. Saudi J Anaesth.

[7] Kohn MD, Sassoon AA, Fernando ND (2016). Classifications in brief: Kellgren-Lawrence classification of osteoarthritis. Clin Orthop Relat Res.

[8] Slattery C (2018). Kweon CY (2018) Classifications in brief: outerbridge classification of chondral lesions. Clin Orthop Relat Res.

[9] Luthringer TA, Blackmore SA, Singh BC, Strauss EJ (2016). The learning curve associated with anteromedial portal drilling in ACL reconstruction. Phys Sportsmed.

[10] Sepúlveda F, Sánchez L, Amy E, Micheo W (2017). Anterior cruciate ligament injury: return to play, function and long-term considerations. Curr Sports Med Rep.

[11] Briggs KK, Steadman JR, Hay CJ, Hines SL (2009). Lysholm score and Tegner activity level in individuals with normal knees. Am J Sports Med.

[12] Padua R, Bondi R, Ceccarelli E, Bondi L, Romanini E, Zanoli G, Campi S (2004). Italian version of the International Knee Documentation Committee Subjective Knee Form: cross-cultural adaptation and validation. Arthroscopy.

[13] van Eck CF, van den Bekerom MP, Fu FH, Poolman RW, Kerkhoffs GM (2013). Methods to diagnose acute anterior cruciate ligament rupture: a meta-analysis of physical examinations with and without anaesthesia. Knee Surg Sports Traumatol Arthrosc.

[14] Loucas M, Loucas R, D'Ambrosi R, Hantes ME (2021). Clinical and radiological outcomes of anteromedial portal versus transtibial technique in ACL reconstruction: a systematic review. Orthop J Sports Med.

[15] van Melick N, van Cingel RE, Brooijmans F, Neeter C, van Tienen T, Hullegie W, Nijhuis-van der Sanden MW (2016). Evidence-based clinical practice update: practice guidelines for anterior cruciate ligament rehabilitation based on a systematic review and multidisciplinary consensus. Br J Sports Med.

[16] Raman IM (2019). Power analysis Elife.

[17] Ventura A, Legnani C, Terzaghi C, Borgo E (2012). Single- and double-bundle anterior cruciate ligament reconstruction in patients aged over 50 years. Arthroscopy.

[18] Fayard JM, Wein F, Ollivier M, Paihle R, Ehlinger M, Lustig S, Panisset JC, French Arthroscopic Society (2019). Factors affecting outcome of ACL reconstruction in over-50-year-olds. Orthop Traumatol Surg Res.

[19] Engler ID, Moradian JR, Pockros BM, Schirmeister CM, Richmond JC, Salzler MJ (2021). Patient-reported outcomes of meniscal repair and meniscectomy in patients 40 years of age and older show similar good results. Knee Surg Sports Traumatol Arthrosc.

[20] Everhart JS, Higgins JD, Poland SG, Abouljoud MM, Flanigan DC (2018). Meniscal repair in patients age 40 years and older: a systematic review of 11 studies and 148 patients. Knee.

[21] Brown MJ, Carter T (2018). ACL allograft: advantages and when to use. Sports Med Arthrosc Rev.

[22] Duchman KR, Lynch TS, Spindler KP (2017). Graft selection in anterior cruciate ligament surgery: who gets what and why?. Clin Sports Med.

[23] Ellis HB, Matheny LM, Briggs KK, Pennock AT, Steadman JR (2012). Outcomes and revision rate after bone-patellar tendon-bone allograft versus autograft anterior cruciate ligament reconstruction in patients aged 18 years or younger with closed physes. Arthroscopy.

[24] Lansdown DA, Riff AJ, Meadows M, Yanke AB, Bach BR (2017). What factors Influence the biomechanical properties of allograft tissue for acl reconstruction? A systematic review. Clin Orthop Relat Res.

[25] Cinque ME, Dornan GJ, Chahla J, Moatshe G, LaPrade RF (2018). High rates of osteoarthritis develop after anterior cruciate ligament surgery: an analysis of 4108 patients. Am J Sports Med.

[26] Struewer J, Frangen TM, Ishaque B, Bliemel C, Efe T, Ruchholtz S, Ziring E (2012). Knee function and prevalence of osteoarthritis after isolated anterior cruciate ligament reconstruction using bone-patellar tendon-bone graft: long-term follow-up. Int Orthop.

[27] van der Hart CP, van den Bekerom MP, Patt TW (2008). The occurrence of osteoarthritis at a minimum of ten years after reconstruction of the anterior cruciate ligament. J Orthop Surg Res.

[28] Cohen M, Amaro JT, Ejnisman B, Carvalho RT, Nakano KK, Peccin MS, Teixeira R, Laurino CF, Abdalla RJ (2007). Anterior cruciate ligament reconstruction after 10 to 15 years: association between meniscectomy and osteoarthrosis. Arthroscopy.

[29] Zaffagnini S, Marcheggiani Muccioli GM, Grassi A, Roberti di Sarsina T, Raggi F, Signorelli C, Urrizola F, Spinnato P, Rimondi E, Marcacci M (2017). Over-the-top ACL reconstruction plus extra-articular lateral tenodesis with hamstring tendon grafts: prospective evaluation with 20-year minimum follow-up. Am J Sports Med.

